# Correction: Manganese mediates antiviral effects by driving an ATM-TBK1 phosphorylation signaling pathway

**DOI:** 10.3389/fimmu.2025.1770238

**Published:** 2026-01-02

**Authors:** Hongyan Sui, Rosana Wiscovitch-Russo, Silvia Cachaco, Jun Yang, Whitney Bruchey, Sylvain Laverdure, Qian Chen, Tomozumi Imamichi

**Affiliations:** 1 Laboratory of Human Retrovirology and Immunoinformatics, Frederick National Laboratory, Frederick, MD, United States; 2 Center for Infectious Disease Research, George Mason University, Manassas, VA, United States

**Keywords:** manganese, ATM, TBK1, phosphorylation, antiviral immunity

Author “Sylvain Laverdure” was erroneously assigned to affiliation “Center for Infectious Disease Research, George Mason University, Manassas, VA, United States”. This affiliation needs to be removed for author “Sylvain Laverdure”.

The **Data Availability statement** was erroneously given as “Publicly available datasets were analyzed in this study. This data can be found here: Microscopy data analyzed in this article have been deposited at GEO previously and are publicly available. Accession number is GSE214181”. The correct **Data Availability statement** is “Publicly available datasets were analyzed in this study. This data can be found here: Microarray data analyzed in this article have been deposited at GEO previously and are publicly available. Accession number is GSE214181”.

