# Supplementary material for: Manganese mediates antiviral effects by driving an ATM -TBK1 phosphorylation signaling pathway
Source: Front Immunol. 2025 Nov 19;16:1708516. doi: 10.3389/fimmu.2025.1708516 (PMC12672510; doi:10.3389/fimmu.2025.1708516)
Supplement: Supplementary Figure 1 — Sequential gating strategy for analysis in IDEAS™ (v6.4) analysis software. (A) Histogram of channel 1 brightfield (BF) gradient RMS of all acquired cells for selection of focused cells. (B) Selection of single cells from focused cells population using a density scatter plot of brightfield area versus brightfield aspect ratio. (C, D) Histogram overlay of intensity of focused single cell subpopulations of AF647 isotype control versus untreated and Mn-treated cells. (E, F) Histogram overlay of intensity of focused single cell subpopulations of AF488 isotype control versus untreated and Mn-treated cells. [file DataSheet1.pdf]

Supplementary material for:

Manganese mediates antiviral effects by driving an ATM -TBK1 phosphorylation signaling pathway

Hongyan Sui[1*], Rosana Wiscovitch-Russo[1], Silvia Cachaco[1,2], Jun Yang[1], Whitney Bruchey[1], Sylvain Laverdure[1], Qian Chen[1], and Tomozumi Imamichi[1]

[1] Laboratory of Human Retrovirology and Immunoinformatics, Frederick National Laboratory, Frederick, Maryland, USA. [2] Current affiliation: Center for Infectious Disease Research, George Mason University, Manassas, Virginia, USA.

*Correspondence:
Hongyan Sui: suih@mail.nih.gov

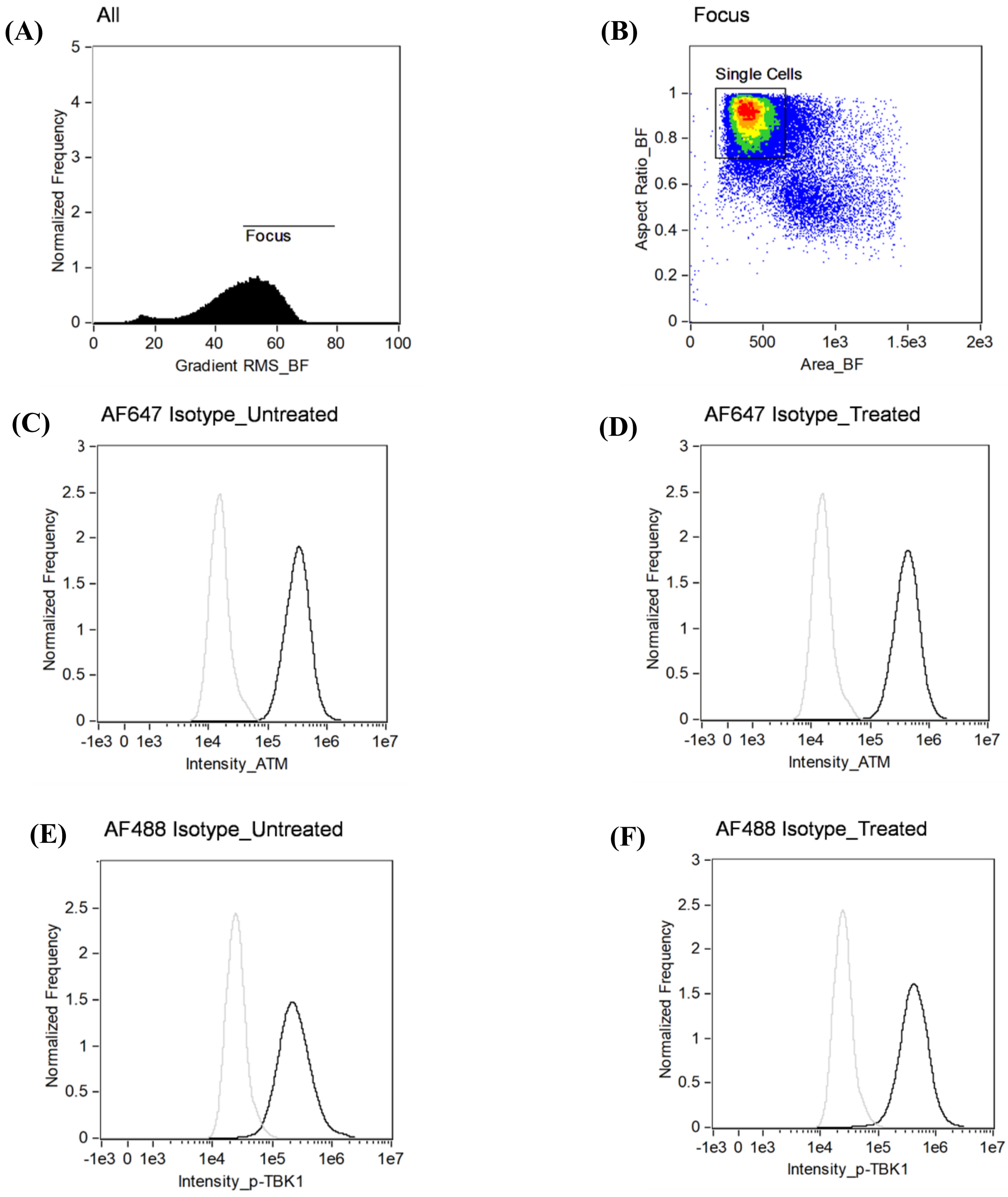

Figure S1. Sequential gating strategy for analysis in IDEAS™ (v6.4) analysis software. (A) Histogram of channel 1 brightfield (BF) gradient RMS of all acquired cells for selection of focused cells. (B) Selection of single cells from focused cells population using a density scatterplot of brightfield area versus brightfield aspect ratio. (C-D) Histogram overlay of intensity of focused single cell subpopulations of AF647 isotype control versus untreated and Mn-treated cells. (E-F) Histogram overlay of intensity of focused single cell subpopulations of AF488 isotype control versus untreated and Mn-treated cells. The data are representative of three independent experiments.

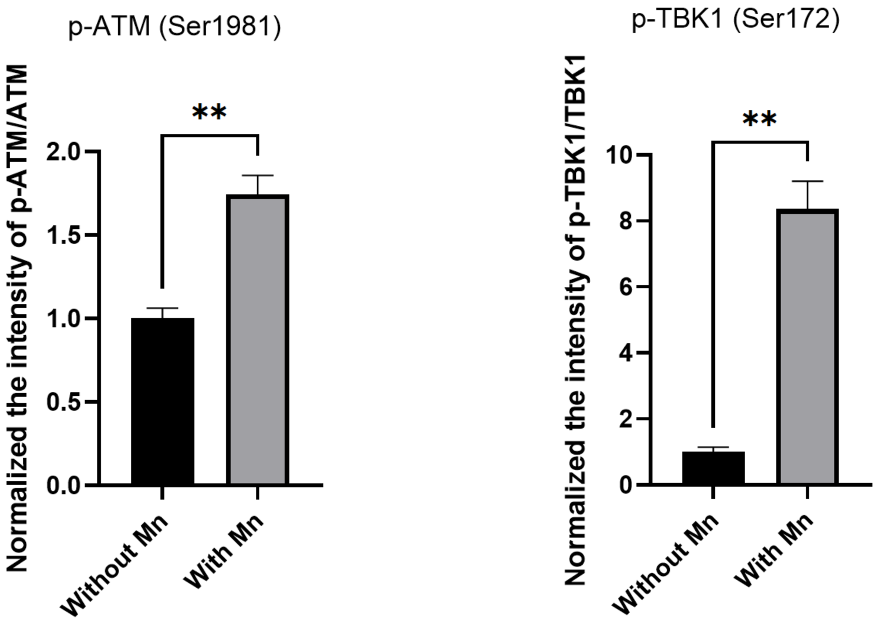

Figure S2. A densitometry analysis was performed according to the data shown in Fig. 4B by Fiji. The intensity of band for p-ATM or p-TBK1 is normalized by total ATM or TBK1, respectively. Data represent at least three independent experiments and are shown as mean ± SD (n=3). A *student's t* test was performed to compare the phosphorylation of ATM and TBK1 under two conditions without Mn vs. with Mn. ** stands for p<0.01.

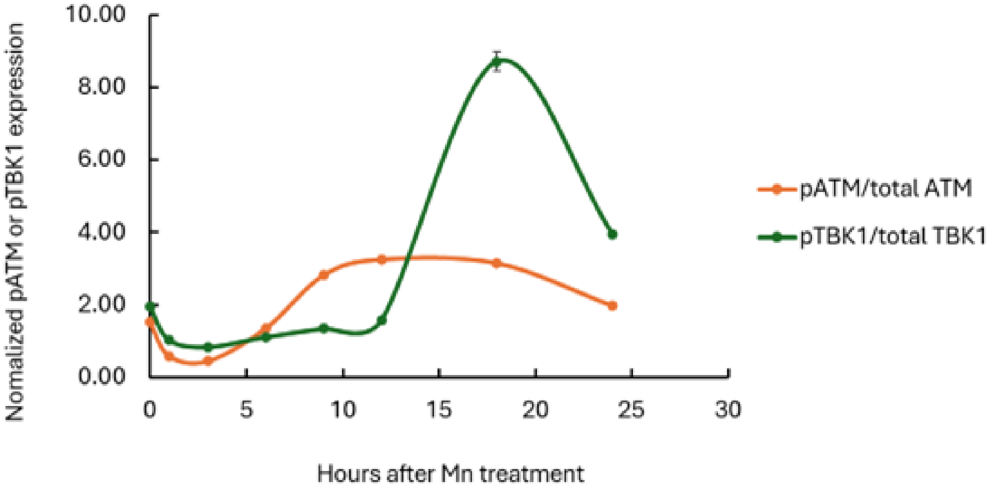

Figure S3. A densitometry analysis was performed according to the data shown in Fig. 4C by Fiji, the intensity of band for p-ATM or p-TBK1 is normalized by total ATM or TBK1, respectively. Data represent at least three independent experiments and are shown as mean ± SD (n=3).

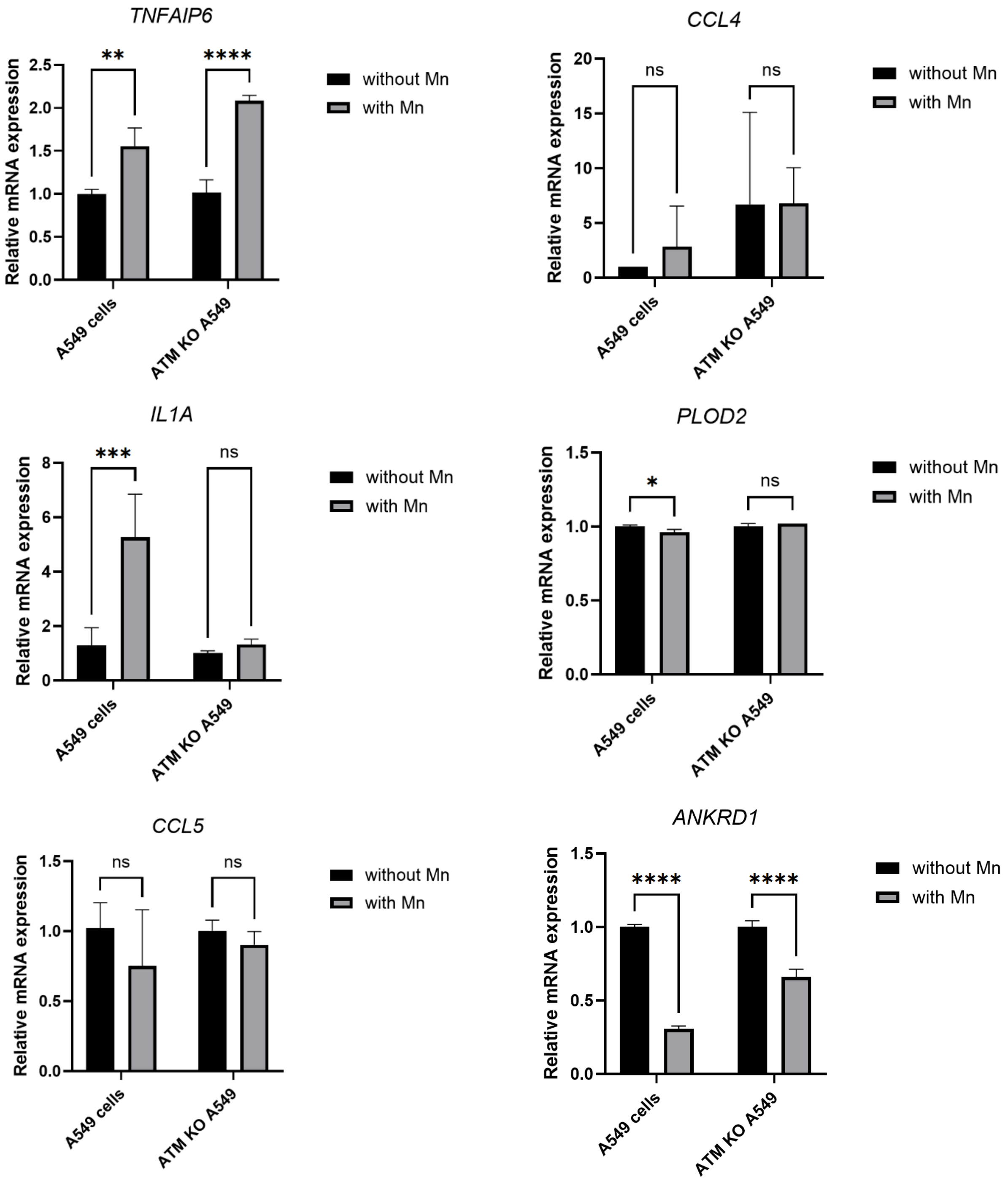

Figure S4. Mn treatment induces far fewer and much lower levels of anti-HIV factors in A549 cells. Total RNA was extracted from with or without Mn-treated A549 or ATM KO A549. Relative mRNA expression of indicated genes was detected using real-time RT-PCR. The values were normalized by GAPDH expression. Data represents three independent experiments and are shown as mean ± SD (n=3). Statistical significance was determined using two-way ANOVA with Bonferroni's multiple comparisons test, $p > 0.05$ (ns), $p<0.05$ (*), $p < 0.001$ (***), and $p < 0.0001$ (****).

Table S1.

A list of antibodies used for western blot, immunofluorescence and Imaging flow cytometry assays. The information about the catalog numbers, suppliers and dilutions is provided.

Antibody	Clone/Isotype	Host	Application	Supplier	Catalog No.
ATM	G-12/IgG1	mouse	WB1:1000; IF, F:1:100	Santa Cruz	Sc-377293
Phos-ATM(Ser1981)	10H11.E12/IgG1	mouse	WB: 1:500	Santa Cruz	Sc-47739
TBK1	D1B4/ IgG	rabbit	WB: 1:1000	Cell signaling	3504S
Phospho-TBK1(Ser172)	D52C2/ IgG	rabbit	WB: 1:1000; IF, F: 1:50	Cell signaling	5483S
β-Actin	AC-74/ IgG2a	mouse	WB: 1:3000	Sigma	A5316
Anti-mouse IgG Antibody (HRP)		sheep	WB: 1:5000	VWR	95017-332
Anti-rabbit IgG (HRP)		donkey	WB: 1:5000	VWR	95017-556
Anti-mouse IgG (H+L), F(ab')2 Fragment (Alexa Fluor® 488 Conjugate)		goat	IF, F:1:1000	Cell signaling	4408S
Anti-rabbit IgG (H+L), F(ab')2 Fragment (Alexa Fluor® 555 Conjugate)		goat	IF, F: 1:1000	Cell signaling	4413S
Anti-rabbit IgG (H+L), F(ab')2 Fragment (Alexa Fluor® 488 Conjugate)		goat	IF, F: 1:1000	Cell signaling	4412S
Anti-mouse IgG (H+L), F(ab')2 Fragment (Alexa Fluor® 647 Conjugate)		goat	IF, F: 1:1000	Cell signaling	4410S
Mouse IgG1 kappa Isotype Control	P3.6.2.8.1	mouse	IF, F: 1:100	Invitrogen	14-4714-82
Rabbit IgG Isotype Control	SP137	rabbit	IF, F: 1:50	Invitrogen	MA5-16384

Application Key: WB- Weston blot; IF-Immunofluorescence; F-Imaging Flow Cytometry

Table S2.

Total 156 genes are listed for Mn upregulated or downregulated anti-HIV factors (fold changes > 2). Group classification corresponds to the cross-referenced databases shown in Figure 6B.

Names	Cross-referenced group	Gene Symbol
CRISPR ISGs Mn vs UT siRNA/shRNA	1	OAS1
CRISPR Mn vs UT siRNA/shRNA	5	ANKRD1
CRISPR Mn vs UT siRNA/shRNA	5	RSAD2
CRISPR Mn vs UT siRNA/shRNA	5	KHDRBS3
CRISPR Mn vs UT siRNA/shRNA	5	MX2
CRISPR Mn vs UT siRNA/shRNA	5	ENC1
ISGs Mn vs UT siRNA/shRNA	7	CCL4
ISGs Mn vs UT siRNA/shRNA	7	CCL5
ISGs Mn vs UT siRNA/shRNA	7	GBP1
ISGs Mn vs UT siRNA/shRNA	7	OASL
ISGs Mn vs UT siRNA/shRNA	7	ISG15
ISGs Mn vs UT siRNA/shRNA	7	IFI44L
ISGs Mn vs UT siRNA/shRNA	7	CD163
CRISPR ISGs Mn vs UT	9	TNFAIP3
CRISPR ISGs Mn vs UT	9	CCL8
CRISPR ISGs Mn vs UT	9	SERPING1
CRISPR ISGs Mn vs UT	9	IFIT2
CRISPR ISGs Mn vs UT	9	IFITM2
CRISPR ISGs Mn vs UT	9	IFIT1
CRISPR ISGs Mn vs UT	9	TNFSF10
CRISPR ISGs Mn vs UT	9	SIGLEC1
CRISPR ISGs Mn vs UT	9	TLR3
CRISPR Mn vs UT	27	LDLR
CRISPR Mn vs UT	27	NFE2L3
CRISPR Mn vs UT	27	ITGAV
CRISPR Mn vs UT	27	TJP2
CRISPR Mn vs UT	27	AKR1B1
CRISPR Mn vs UT	27	AEN
CRISPR Mn vs UT	27	LAT
CRISPR Mn vs UT	27	HIP1
CRISPR Mn vs UT	27	SLC16A3
CRISPR Mn vs UT	27	ENO1
CRISPR Mn vs UT	27	HYOU1
CRISPR Mn vs UT	27	MTF1
CRISPR Mn vs UT	27	USP11
CRISPR Mn vs UT	27	MFAP5
CRISPR Mn vs UT	27	UACA
CRISPR Mn vs UT	27	PSIP1
CRISPR Mn vs UT	27	UTRN
CRISPR Mn vs UT	27	RAVER2
CRISPR Mn vs UT	27	MXD4
CRISPR Mn vs UT	27	HIST1H3I
CRISPR Mn vs UT	27	FLI1

CRISPR Mn vs UT	27	KAT2A
CRISPR Mn vs UT	27	MSH2
CRISPR Mn vs UT	27	JUP
CRISPR Mn vs UT	27	NUP210
CRISPR Mn vs UT	27	ASRGL1
CRISPR Mn vs UT	27	ITGA4
ISGs Mn vs UT	49	TNFAIP6
ISGs Mn vs UT	49	GCH1
ISGs Mn vs UT	49	CD80
ISGs Mn vs UT	49	CD274
ISGs Mn vs UT	49	C15orf48
ISGs Mn vs UT	49	SERPINE1
ISGs Mn vs UT	49	LAMP3
ISGs Mn vs UT	49	GEM
ISGs Mn vs UT	49	ADM
ISGs Mn vs UT	49	DDIT4
ISGs Mn vs UT	49	BIRC3
ISGs Mn vs UT	49	PMAIP1
ISGs Mn vs UT	49	DUSP5
ISGs Mn vs UT	49	IDO1
ISGs Mn vs UT	49	RGS1
ISGs Mn vs UT	49	ABTB2
ISGs Mn vs UT	49	NFIL3
ISGs Mn vs UT	49	ATF3
ISGs Mn vs UT	49	PDK1
ISGs Mn vs UT	49	ABLIM3
ISGs Mn vs UT	49	CDKN1A
ISGs Mn vs UT	49	APOL3
ISGs Mn vs UT	49	HK2
ISGs Mn vs UT	49	IL1RN
ISGs Mn vs UT	49	SERPINB9
ISGs Mn vs UT	49	IRF1
ISGs Mn vs UT	49	CCL2
ISGs Mn vs UT	49	ARG2
ISGs Mn vs UT	49	TAP1
ISGs Mn vs UT	49	OPTN
ISGs Mn vs UT	49	TAP2
ISGs Mn vs UT	49	RNF24
ISGs Mn vs UT	49	TNFRSF10A
ISGs Mn vs UT	49	IFI16
ISGs Mn vs UT	49	IFNGR1

ISGs Mn vs UT	49	MT1F
ISGs Mn vs UT	49	UBA7
ISGs Mn vs UT	49	GCA
ISGs Mn vs UT	49	MAP3K5
ISGs Mn vs UT	49	FAM46A
ISGs Mn vs UT	49	HESX1
ISGs Mn vs UT	49	TRIM14
ISGs Mn vs UT	49	PADI2
ISGs Mn vs UT	49	FCGR1A
ISGs Mn vs UT	49	SECTM1
ISGs Mn vs UT	49	MS4A4A
ISGs Mn vs UT	49	STAP1
ISGs Mn vs UT	49	HPSE
ISGs Mn vs UT	49	TLR7
Mn vs UT siRNA/shRNA	58	IL1A
Mn vs UT siRNA/shRNA	58	PLOD2
Mn vs UT siRNA/shRNA	58	JAG1
Mn vs UT siRNA/shRNA	58	HCAR3
Mn vs UT siRNA/shRNA	58	ZSWIM4
Mn vs UT siRNA/shRNA	58	GCLM
Mn vs UT siRNA/shRNA	58	ITGB8
Mn vs UT siRNA/shRNA	58	MET
Mn vs UT siRNA/shRNA	58	CRIM1
Mn vs UT siRNA/shRNA	58	RRAS2
Mn vs UT siRNA/shRNA	58	ETS2
Mn vs UT siRNA/shRNA	58	SLC2A3
Mn vs UT siRNA/shRNA	58	SSTR2
Mn vs UT siRNA/shRNA	58	ARRDC4
Mn vs UT siRNA/shRNA	58	DNAJC6
Mn vs UT siRNA/shRNA	58	TAGLN2
Mn vs UT siRNA/shRNA	58	RUSC2
Mn vs UT siRNA/shRNA	58	PELI1
Mn vs UT siRNA/shRNA	58	NR4A2
Mn vs UT siRNA/shRNA	58	SEC61G
Mn vs UT siRNA/shRNA	58	NFKB1
Mn vs UT siRNA/shRNA	58	CLGN
Mn vs UT siRNA/shRNA	58	AFF2
Mn vs UT siRNA/shRNA	58	RAP1B
Mn vs UT siRNA/shRNA	58	CLDN12
Mn vs UT siRNA/shRNA	58	PSMA3
Mn vs UT siRNA/shRNA	58	NENF

Mn vs UT siRNA/shRNA	58	RYBP
Mn vs UT siRNA/shRNA	58	PCSK6
Mn vs UT siRNA/shRNA	58	PSME2
Mn vs UT siRNA/shRNA	58	SPATA18
Mn vs UT siRNA/shRNA	58	NRIP3
Mn vs UT siRNA/shRNA	58	MT2A
Mn vs UT siRNA/shRNA	58	LYPLAL1
Mn vs UT siRNA/shRNA	58	NREP
Mn vs UT siRNA/shRNA	58	NOTCH2NL
Mn vs UT siRNA/shRNA	58	ZC3H6
Mn vs UT siRNA/shRNA	58	EVI2A
Mn vs UT siRNA/shRNA	58	NBPF1
Mn vs UT siRNA/shRNA	58	SNRPN
Mn vs UT siRNA/shRNA	58	XYLT1
Mn vs UT siRNA/shRNA	58	ATP6V0A1
Mn vs UT siRNA/shRNA	58	PER3
Mn vs UT siRNA/shRNA	58	TMEM117
Mn vs UT siRNA/shRNA	58	SASH3
Mn vs UT siRNA/shRNA	58	ACACB
Mn vs UT siRNA/shRNA	58	CCNG2
Mn vs UT siRNA/shRNA	58	STAB1
Mn vs UT siRNA/shRNA	58	MAML3
Mn vs UT siRNA/shRNA	58	KIAA0922
Mn vs UT siRNA/shRNA	58	NUDT3
Mn vs UT siRNA/shRNA	58	CD209
Mn vs UT siRNA/shRNA	58	CMAHP
Mn vs UT siRNA/shRNA	58	SLC46A1
Mn vs UT siRNA/shRNA	58	MRC1
Mn vs UT siRNA/shRNA	58	KCNJ5
Mn vs UT siRNA/shRNA	58	IQCK
Mn vs UT siRNA/shRNA	58	SLC6A16
